# Performance evaluation of a prototype rapid diagnostic test for combined detection of *gambiense* human African trypanosomiasis and malaria

**DOI:** 10.1371/journal.pntd.0008168

**Published:** 2020-04-06

**Authors:** Crispin Lumbala, Enock Matovu, Hakim Sendagire, Anne J. N. Kazibwe, Joris L. Likwela, Hypolite Muhindo Mavoko, Simon Kayembe, Pascal Lutumba, Sylvain Biéler, Jean-Pierre Van Geertruyden, Joseph M. Ndung’u

**Affiliations:** 1 Disease Control Directorate, Ministry of Public Health, Democratic Republic of the Congo; 2 Global Health Institute, Faculty of Medicine and Health Sciences, University of Antwerp, Antwerp, Belgium; 3 College of Veterinary Medicine, Animal Resources and Biosecurity, Makerere University, Kampala, Uganda; 4 College of Health Sciences, Makerere University, Kampala, Uganda; 5 Public Health Department, Faculty of Medicine and Pharmacy, University of Kisangani, Kisangani, Democratic Republic of the Congo; 6 Kinshasa University, Kinshasa, Democratic Republic of the Congo; 7 Foundation for Innovative New Diagnostics (FIND), Geneva, Switzerland; Institute of Tropical Medicine, BELGIUM

## Abstract

**Background:**

Malaria is endemic in all regions where *gambiense* or *rhodesiense* human African trypanosomiasis (HAT) is reported, and both diseases have similarities in their symptomatology. A combined test could be useful for both diseases and would facilitate integration of the screening for *gambiense* HAT (gHAT) and malaria diagnosis. This study aimed to evaluate a combined prototype rapid diagnostic test (RDT) for gHAT and malaria.

**Methods:**

Blood samples were collected in the Democratic Republic of the Congo and in Uganda to evaluate the performance of a prototype HAT/Malaria Combined RDT in comparison to an individual malaria RDT based on *Plasmodium falciparum* (*P*.*f*.) Histidine Rich Protein II (HRP-II or HRP2) antigen (SD BIOLINE Malaria Ag *P*.*f*. RDT) for malaria detection and an individual gHAT RDT based on recombinant antigens, the SD BIOLINE HAT 2.0 RDT for HAT screening. Due to the current low prevalence of gHAT in endemic regions, the set of blood samples that were collected was used to evaluate the specificity of the RDTs for gHAT, and additional archived plasma samples were used to complete the evaluation of the HAT/Malaria Combined RDT in comparison to the HAT 2.0 RDT.

**Results:**

Frozen whole blood samples from a total of 486 malaria cases and 239 non-malaria controls, as well as archived plasma samples from 246 gHAT positive and 246 gHAT negative individuals were tested. For malaria, the sensitivity and specificity of the malaria band in the HAT/Malaria Combined RDT were 96.9% (95% CI: 95.0–98.3) and 97.1% (95% CI: 94.1–98.8) respectively. The sensitivity and specificity of the SD BIOLINE malaria Ag *P*.*f*. RDT were 97.3% (95% CI: 95.5–98.6) and 97.1% (95% CI: 94.1–98.8) respectively. For gHAT, using archived plasma samples, the sensitivity and specificity were respectively 89% (95% CI: 84.4–92.6) and 93.5% (95% CI: 89.7–96.2) with the HAT/Malaria Combined RDT, and 88.2% (95% CI: 83.5–92) and 94.7% (95% CI: 91.1–97.2) with the HAT 2.0 RDT. Using the whole blood samples that were collected during the study, the specificity of the HAT/Malaria Combined RDT for gHAT was 95.8% (95% CI: 94.3–97.0).

**Conclusion:**

The HAT/Malaria Combined prototype RDT was as accurate as the individual malaria or gHAT RDTs. The HAT/Malaria Combined prototype RDT is therefore suitable for both malaria diagnosis and gHAT screening. However, there is a need to assess its accuracy using fresh samples in prospective clinical trials.

## Author summary

The annual number of reported cases of human African trypanosomiasis (HAT), also known as sleeping sickness (SS), is currently below 1,000 cases worldwide. The Democratic Republic of the Congo (DRC), the most affected country, and Uganda, which shares a border with DRC, are both endemic for *gambiense* HAT (gHAT). The main strategy to control gHAT is screening of at-risk individuals, followed by diagnosis and treatment of confirmed cases. However, this strategy and even the passive screening as currently implemented become less efficient with declining incidence, justifying innovative strategies to efficiently detect the remaining cases. All areas where gHAT occurs are also endemic for malaria, presenting an opportunity to integrate gHAT screening activities within malaria control activities. This integration is warranted by the fact that in early disease stage, gHAT patients present with signs and symptoms strikingly similar to those of malaria. In order to use malaria diagnosis as an entry point to screen for gHAT, Standard Diagnostics (SD), Republic of Korea (now Abbott Diagnostics, Korea Inc–ADK) made a Combined prototype RDT for both malaria and gHAT, expected to be as accurate as the individual gHAT and malaria RDTs. In this study, we evaluated the accuracy of the Combined prototype RDT using whole blood samples collected in Uganda and DRC, and archived plasma samples collected in DRC, Angola and Central African Republic. We found that the Combined prototype performs just as well as individual RDTs.

## Introduction

**Fig 1:**
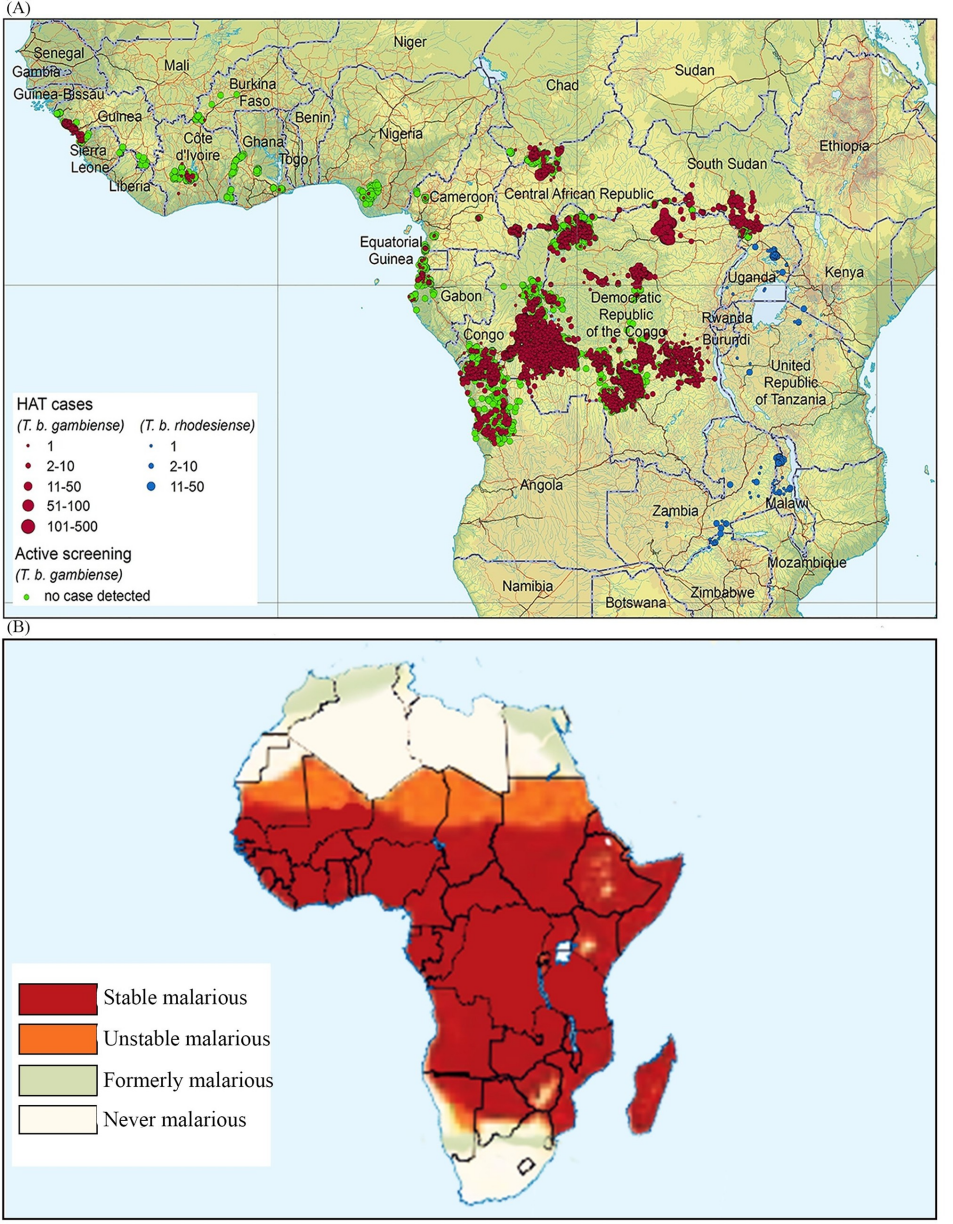
(A) Distribution of human African trypanosomiasis cases reported between 2010 and 2014 [13] and (B) African map of malaria prevalence in 2009 and before (reproduced from “World-map-of-past-and-current-malaria-prevalence-world-development-report-2009.png” file, https://images.app.goo.gl/YYomgUC8fC9ZjTFXA).

Malaria transmission is ongoing in all regions where human African trypanosomiasis (HAT, also known as sleeping sickness), is endemic. However, the opposite is not true [1–4]. This geographic overlap between malaria and HAT (Fig 1) provides a unique opportunity for an integrated control approach for both diseases in the areas where they overlap. Indeed, both diseases need accurate diagnostic tools to guide treatment and control [4–6].

HAT is a vector-borne disease transmitted in sub-Saharan Africa through the bite of several species and subspecies of tsetse flies of the genus *Glossina*. HAT is caused by two subspecies of *Trypanosoma brucei (T*.*b*.*)* namely *T*.*b*. *gambiense* and *T*.*b*. *rhodesiense*. *Trypanosoma b*. *gambiense* is responsible for a chronic form of the disease, while *T*.*b*. *rhodesiense* causes a more acute disease form. *Rhodesiense* HAT (rHAT) is found in eastern and southern Africa. It is a zoonotic disease that only accidentally involves human beings. On the other hand, *gambiense* HAT (gHAT) is endemic in central and western Africa and represents >98% of all HAT cases [4, 7, 8].

Thirty-six African countries have historically been endemic for HAT, of which 24 are endemic for *gambiense* HAT (referred to as HAT or gHAT), and 13 for *rhodesiense* HAT (referred to as rHAT). Uganda is endemic for both forms. Among the 24 countries affected by *T*.*b*. *gambiense*, Angola, the Democratic Republic of the Congo (DRC), South Sudan, Chad, the Central African Republic (CAR), the Republic of Congo, Guinea and Uganda represent 98% of all reported gHAT cases [9]. The DRC reported up to 85% of all cases between 2012 and 2016 [8, 10].

Malaria is a vector-borne disease caused by *Plasmodium* spp. In 2018, a total of 228 million cases of malaria were reported compared to 216 million in 2016. If left untreated, malaria may result in severe complications and lead to death. In 2018, 405,000 deaths due to the disease were reported globally compared to 445,000 in 2016. The African region continues to account for more than 90% of malaria cases and deaths worldwide. DRC is second the most widely affected country in the world, after Nigeria; it accounted for 12% of reported cases and 11% of deaths in 2018 [11, 12].

Patients with malaria or early stage HAT often present with similar symptoms, such as headache, flu-like symptoms, malaise, joint pains, fever and chills. Diagnostic tests are therefore needed to differentiate these diseases and ensure early HAT treatment to prevent complications, such as irreversible sequelae due to invasion of the central nervous system (CNS) by parasites [4, 5, 9, 14–17]. Delayed diagnosis and treatment of both diseases also promotes further disease transmission. As such, diagnostic tools for both diseases play a key role in control and elimination efforts. Whereas HAT control programs are presently targeting elimination [18, 19], malaria programs in HAT endemic countries target disease control as malaria is still highly endemic in these areas [20]. The substantial decrease in HAT prevalence in recent decades has led to a well-known public health paradox, which is that the few remaining cases and/or carriers are more difficult to identify, cost more money and intervention for HAT control activities become less efficient, with consequences that donors could stop funding for HAT. This situation hinders elimination and could lead to HAT re-emergence. Therefore, there is a need for innovative and cost-effective strategies to identify the last HAT cases in order to reach and sustain elimination. Such strategies should consider disease spread, preparedness, involvement of health care facilities in the elimination process and opportunities in the overall health care system [2, 21]. As HAT occurs in areas that are highly endemic for malaria, HAT elimination programs could benefit from the well-established and omnipresent malaria control networks by introducing an effective and affordable diagnostic tool for HAT that would be used in an integrated manner for malaria diagnosis. This would allow for a sustained screening for HAT in a subset of the population living in HAT and malaria co-endemic areas.

Today, in addition to microscopy, the diagnostic test that is recommended for malaria in most sub-Saharan African countries is a rapid diagnostic test (RDT) based on *Plasmodium falciparum* (*P*.*f*.) Histidine Rich Protein II (HRP-II or HRP2) antigen (Ag) [6]. For HAT screening, the most widely used tool has been the Card Agglutination Test for Trypanosomiasis (CATT), which has played an important role in reducing the HAT burden. However, CATT is difficult to implement in peripheral health facilities because of the need for a cold chain for reagent storage, equipment requiring electricity and lack of a single test format [18]. To address these challenges, Standard Diagnostics, Inc. (SD, Geonggi-do, South-Korea), now Abbott Diagnostics Inc, Korea, and Coris Bioconcept (Gembloux, Belgium) have each developed RDTs for HAT screening [22–25]. These tests have been adopted and recommended by the World Health Organization (WHO) [4, 24].

Since areas that are endemic for HAT are also endemic for malaria, both HAT and malaria RDTs could be used on the same patients. Therefore, with support from the Foundation for Innovative New Diagnostics (FIND, Geneva, Switzerland), SD developed a prototype test that combines HAT screening and malaria diagnosis in the same cassette. Screening for HAT is based on antibody detection, and seropositive suspects have to undergo confirmatory testing by microscopy before treatment can be initiated. In contrast, malaria RDTs are based on antigen detection, and a positive result is usually sufficient for a decision to start treatment, although this depends on the national guidelines. A prototype SD BIOLINE HAT/Malaria test was made by combining the SD BIOLINE HAT 2.0 RDT [24] and the SD BIOLINE Malaria Ag *P*.*f*. RDT. This combined test would be targeted for use in the diagnosis of malaria, with a comparative advantage that screening for HAT is done simultaneously. This would enable detection and treatment of residual cases of HAT during malaria testing, and thus contribute to efforts to prevent re-emergence of HAT and sustain its elimination.

We report here the accuracy of the SD BIOLINE HAT/Malaria Combined prototype RDT (referred to as Combo RDT) in comparison with HAT and Malaria RDTs in individual formats (referred to as HAT 2.0 RDT and SD Malaria *P*.*f*. RDT).

## Methods

### Ethics statement

All diagnostic tests, data entry and data management were conducted according to Standard Operating Procedures (SOP).

The project was carried out in conformity with the Declaration of Helsinki. The sites were appropriate health facilities with staff trained to enrol participants and collect blood samples under good clinical and laboratory practices (GCLP) conditions. The study protocol was approved by the Ethics Committee in DRC (Ethics Committee of the School of Public Health, University of Kinshasa) and in Uganda (The Uganda National Council for Science and Technology). Before inclusion, written informed consent was obtained from participants or legal guardians of minors.

### Study design and sites

#### Malaria assessment

**Fig 2:**
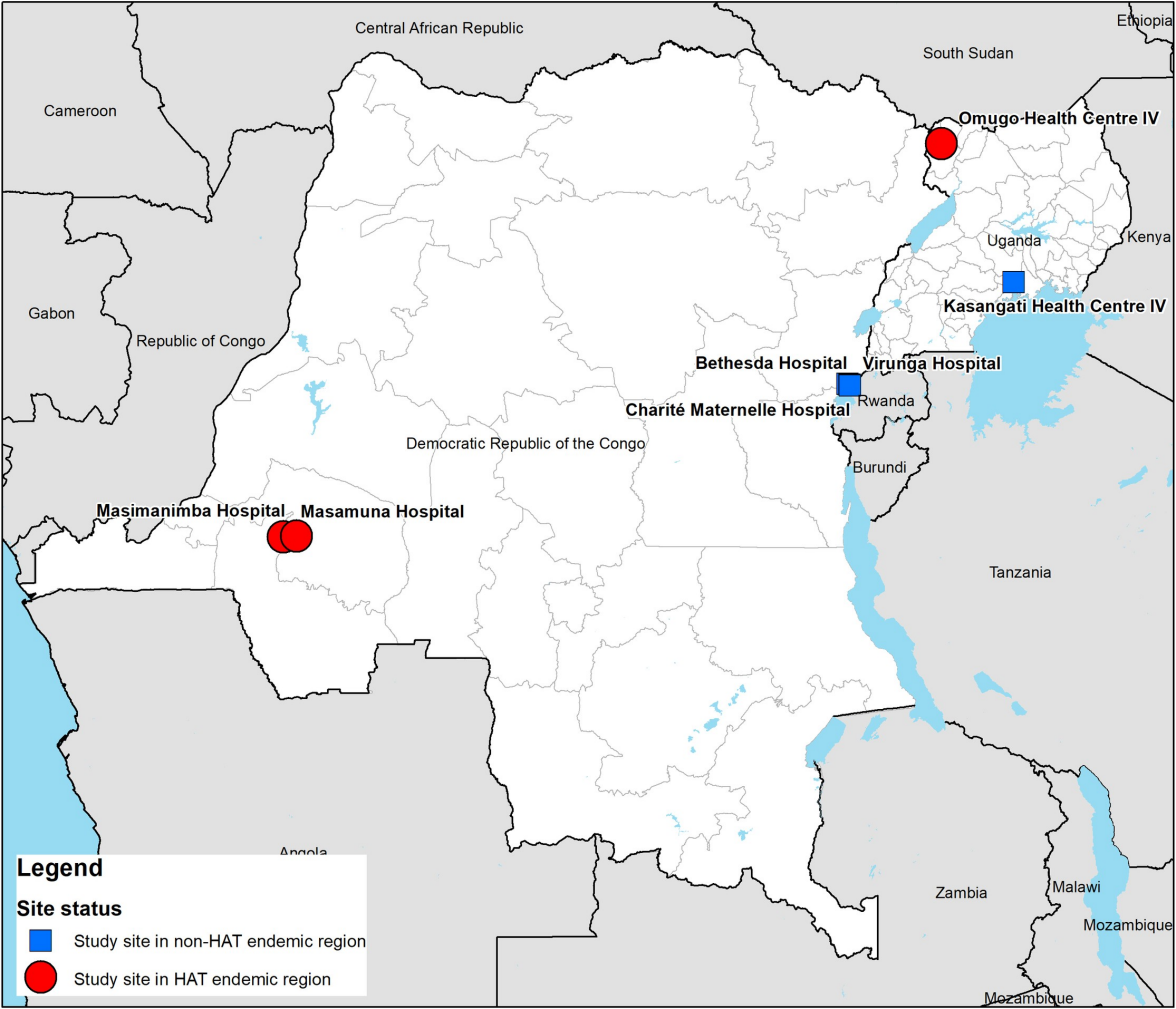
Whole blood sample collection sites.

To evaluate the accuracy of malaria diagnosis, samples of whole blood were collected from 7 study sites in the DRC and in Uganda, including 3 sites located in HAT endemic regions and 4 in regions where HAT is not endemic (Fig 2). The 3 study sites in HAT endemic regions were Omugo Health Centre IV in Uganda (Arua district), as well as Masamuna and Masimanimba Hospitals in the DRC (Kwilu province, part of former Bandundu province). The 4 study sites located in regions that are not endemic for HAT were Kasangati Health Centre IV in Uganda (Wakiso district), as well as Bethesda, Virunga and Charité Maternelle Hospitals in the DRC (Nord Kivu province).

#### HAT assessment

In view of the current low prevalence of HAT in endemic regions, evaluation of the accuracy of the prototype RDT for HAT screening was done on plasma samples that had been obtained from HAT endemic countries (Angola, CAR and DRC) as part of earlier research projects, and archived at Makerere University (Kampala, Uganda). The whole blood samples that were collected during the current study were used to evaluate the specificity of the prototype RDT for HAT.

### Description of study population

#### Malaria assessment

Patients presenting themselves at health facilities with symptoms suggestive of malaria or HAT (such as nausea, vomiting, anorexia, flu, weakness, fever, headache, neck pain, body ache, joint pain, itching, sleeping, speech or movement disturbances, weight loss, etc), aged at least 6 months (in DRC) or at least 6 years (in Uganda), and who provided informed consent, were enrolled for sample collection and characterization. Patients with severe anaemia from whom blood collection was not possible, or whose condition was such that they could not give informed consent were excluded from the study.

Microscopy is currently the common test used as gold standard diagnostic for malaria and is affordable at the level of health facilities. Its shortcomings include the requirement for well-trained and experienced microscopists, rigorous maintenance of equipment, good quality supplies, and that patients with low parasite densities are often missed. Polymerase Chain Reaction (PCR) is more accurate and can detect sub-microscopic malaria infection, but it requires complex equipment, is time consuming and costly [26, 27]. Thus, the reference test (RT) for malaria in this study was microscopy (performed in the field on fresh blood samples) and PCR (performed at Makerere University on stored samples). A sample was considered positive for malaria when it was positive by both microscopy and PCR. It was considered negative if negative by both tests. The sample was excluded if microscopy and PCR results were discordant.

For microscopy, a thick and a thin blood smear were prepared from each participant. The thin smear was fixed with methanol, and all the blood smears stained with 10% Giemsa for 10 minutes. Thick blood smears were examined by microscopy for the presence of asexual parasites and gametocytes. Parasite densities were calculated as described by Adu-Gyasi *et al* (2012) [28] by counting the number of asexual parasites per 200 leucocytes (or per 500, if the count was <10 asexual parasites/200 leucocytes) and adjusting based on the number of white blood cells (WBCs). WBCs were counted manually using a Neubauer haemocytometer. A blood smear was considered negative when examination of 100 fields at X1000 did not reveal asexual parasites. In the case of a positive thick smear, the corresponding thin blood smear was examined to determine the parasite species.

Nested PCR was used to detect malaria parasites and to identify the species. Amplification of the 18S rRNA gene was performed using methods and primers described previously [29–31]. It included a primary PCR test using generic primers for *Plasmodium* spp., and a secondary PCR test performed on the product of the primary PCR, using primers specific for *P*. *falciparum*. The secondary PCR product was visualized on an agarose gel and a qualitative result indicating the presence or absence of a band of the expected size recorded.

#### HAT assessment

For HAT, stored plasma samples collected in Angola and Central African Republic (CAR) had been pre-selected by CATT, and the ones collected in DRC had been pre-selected by CATT, the SD BIOLINE HAT RDT and/or the SD BIOLINE HAT 2.0 RDT (referred to as HAT RDT and HAT 2.0 RDT respectively) [22–24]. Confirmatory diagnosis of HAT was based on a Composite Reference Standard (CRS), including microscopy of lymph node aspirate, blood examination by the micro-Haematocrit Centrifugation Technique (mHCT) and/or mini-anion exchange centrifugation technique (mAECT), and CSF examination using the modified single centrifugation (MSC) technique [4]; mAECT was unavailable for CAR evaluations. Samples were considered HAT positive (i.e. from HAT cases) if collected from persons from whom trypanosomes were identified by any of the microscopy methods in the CRS. Plasma samples collected from persons with negative HAT serology and parasitological tests and no history of HAT were considered as controls. Participants enrolled in areas that were not HAT endemic were assumed to be negative for HAT and considered as non-HAT controls.

The whole blood samples that were collected during the current study were preselected using the HAT RDT and HAT 2.0 RDT as screening tests in the DRC, and using the HAT RDT in Uganda. Confirmatory diagnosis of HAT was based on the CRS described above. HAT case and non-HAT control participants were considered as described above.

#### Sample size

No formal sample size calculations were performed for HAT or malaria assessments.

The target was to collect fresh whole blood samples from 500 malaria case and 500 non-malaria control participants (no target numbers were set for HAT cases or non-HAT control participants). In addition, archived plasma samples from 250 HAT cases and 250 non-HAT controls were used. This was expected to provide information that would be strong enough to evaluate the prototype combo RDT, and inform decisions to start prospective performance evaluation studies in the field.

Whole blood samples collected from malaria cases and non-malaria control participants were used to assess the malaria accuracy and HAT specificity of RDTs, while archived plasma samples were used to assess HAT accuracy.

### HAT and malaria RDTs

The SD BIOLINE Malaria Ag *P*.*f*. test (SD Malaria *P*.*f*. RDT), the SD BIOLINE HAT 2.0 RDT (HAT 2.0 RDT), and the prototype SD BIOLINE HAT/Malaria Combined test (Combo RDT) are all cassette-format RDTs developed by SD. They include one control band (C band) and one or two test bands for HAT and/or malaria.

The SD Malaria *P*.*f*. RDT includes one test band for the qualitative detection of the *P*. *falciparum* HRP-II antigen in human whole blood.

The HAT 2.0 RDT includes 2 test bands to detect antibodies against two trypanosome antigens (recombinant ISG65 and recombinant VSG LiTat 1.5) [24].

The Combo RDT includes two test bands: one to detect the *P*. *falciparum* HRP-II antigen (“*P*.*f*.” or “malaria” band) and the other to detect antibodies against two trypanosome antigens (recombinant ISG65 and recombinant VSG LiTat 1.5) (“HAT” band).

#### Identifiers of proteins cited in this study

*Plasmodium falciparum* histidine rich protein (HRP-II), accession P05227.1 (UniProtKB/Swiss-Prot); *T*. *brucei* ISG65, accession XP_951587 (GenPept); and *T*. *brucei* VSG LiTat1.5, accession ABX55936 (GenPept).

### Collection and use of fresh whole blood samples

#### Malaria assessment

To evaluate the accuracy of the RDTs, 4 ml of venous blood were collected by trained staff of the health facility, into tubes containing heparin as anticoagulant. The blood samples were subdivided into two portions and blinded by an independent technician prior to testing. One portion of each sample was used onsite to perform the following tests and procedures: SD BIOLINE Malaria Ag *P*.*f*.*/Pan* or Ag *P*.*f*. RDT, microscopy procedures for malaria (regardless of onsite malaria RDT results). From the second portion of blood, 4 aliquots of 500μl each were prepared and frozen in liquid nitrogen and shipped to Makerere University for malaria PCR analysis, and testing with both the SD Malaria *P*.*f*. RDT and the prototype Combo RDT.

Malaria RDT results obtained on site were used to manage malaria according to the country’s guidelines.

#### HAT assessment

The collected portions of blood described above were also used for HAT assessments as follows:

- one portion was tested onsite (2 ml) with HAT RDT and/or HAT 2.0 RDT and subjected to HAT parasitology testing (mHCT and mAECT).
- the aliquots shipped to Makerere University were also tested with the prototype Combo RDT (HAT band).

The RDT results obtained onsite were used to guide confirmatory testing by microscopy, according to the HAT diagnostic algorithm.

### Evaluation of RDTs at Makerere University

#### Malaria assessment

Blood samples shipped frozen on dry ice to Makerere University were thawed at room temperature and mixed to ensure homogeneity. All samples were labelled with blinding codes.

To evaluate the accuracy of malaria diagnosis, 5 μl of whole blood was applied to the SD Malaria *P*.*f*. RDT and to the Combo RDT (malaria band), according to the manufacturer’s instructions. The procedure for performing the Combo RDT is the same as that for the SD Malaria *P*.*f*. RDT, except that the former includes two test lines (one for HAT and another for malaria).

#### HAT assessment

To evaluate the accuracy of HAT diagnosis, using archived plasma samples, an aliquot of 15 μl of plasma was diluted by adding 15 μl of human blood, freshly collected from a volunteer. This was done in order to ensure that the volume to pipette for RDT testing would not be too small. The sample of whole blood from the volunteer was first checked with each of the 3 RDTs being evaluated, to ensure that it did not give any positive HAT or malaria result. Volumes of 5 μl and 20 μl were then transferred from this 30 μl aliquot and applied to the Combo RDT (HAT band) and to the HAT 2.0 RDT, respectively.

Frozen blood samples were tested with the combo RDT as described above, and the result of HAT band recorded.

### Data management and statistical analysis

Participant information and test results at the sample collection sites were recorded on paper case report forms (CRF). The CRFs were transferred to “Programme National de Lutte contre la Trypanosomiase Humaine Africaine” (PNLTHA) in Kinshasa (DRC) or to Makerere University (Uganda) for double data entry using a web-based clinical data management platform (Open Clinica). Data were analysed with Stata SE 15.1 software.

Sensitivity and specificity were calculated for each test as the percentage of positive tests among cases and the percentage of negative tests among controls respectively. Accuracy was assessed by calculating Youden’s index [32]. Concordance between different RDTs was also assessed by calculating the corresponding Cohen’s kappa factor. We evaluated sensitivity, specificity and Youden’s index using 1^st^ reader RDT results, and we assessed the inter-agreement between two independent RDT readers using Cohen’s kappa factor.

Positive and negative predictive values (PPV and NPV) were estimated based on the sensitivity and specificity using HAT prevalence values of 0.01%, 0.1%, 1% and 2%. The HAT prevalence in the population through active screening is currently less than 1%. However, in passive screening this proportion could be up to 1% or 2%, especially among patients that would be preselected based on signs and symptoms indicative of HAT. False discovery rate (FDR) and false omission rate (FOR), easy translation of PPV and NPV by a surveillance programme, could be estimated from PPV and NPV as described below. Based on the DRC National Malaria Control Program (Programme National de Lutte contre le Paludisme—PNLP) annual report for 2016, about 70% of malaria suspects tested positive with malaria RDTs among people attending health facilities. We evaluated and reported the PPV and NPV considering a pre-test probability in health facility setting of 70%.

PPV = sensitivity x prevalence / (sensitivity x prevalence) + (1 –specificity) x (1 –prevalence)

NPV = (specificity x (1 –prevalence)) / ((1 –sensitivity) x prevalence) + (specificity x (1 –prevalence))

FDR = 1 –PPV

FOR = 1 –NPV

## Results

### Malaria

#### Enrolment

Among 725 participants enrolled, 486 were malaria cases and 239 non-malaria controls. Of the total participants, 459 (including 340 malaria cases) were enrolled in HAT endemic regions, while 266 (146 malaria cases) were from non-HAT regions. The median age was 24 years with an interquartile range (IQR) of 17 years (18–35 years old). The proportion of females was 61.9% (95% CI: 58.3–65.4).

**Fig 3:**
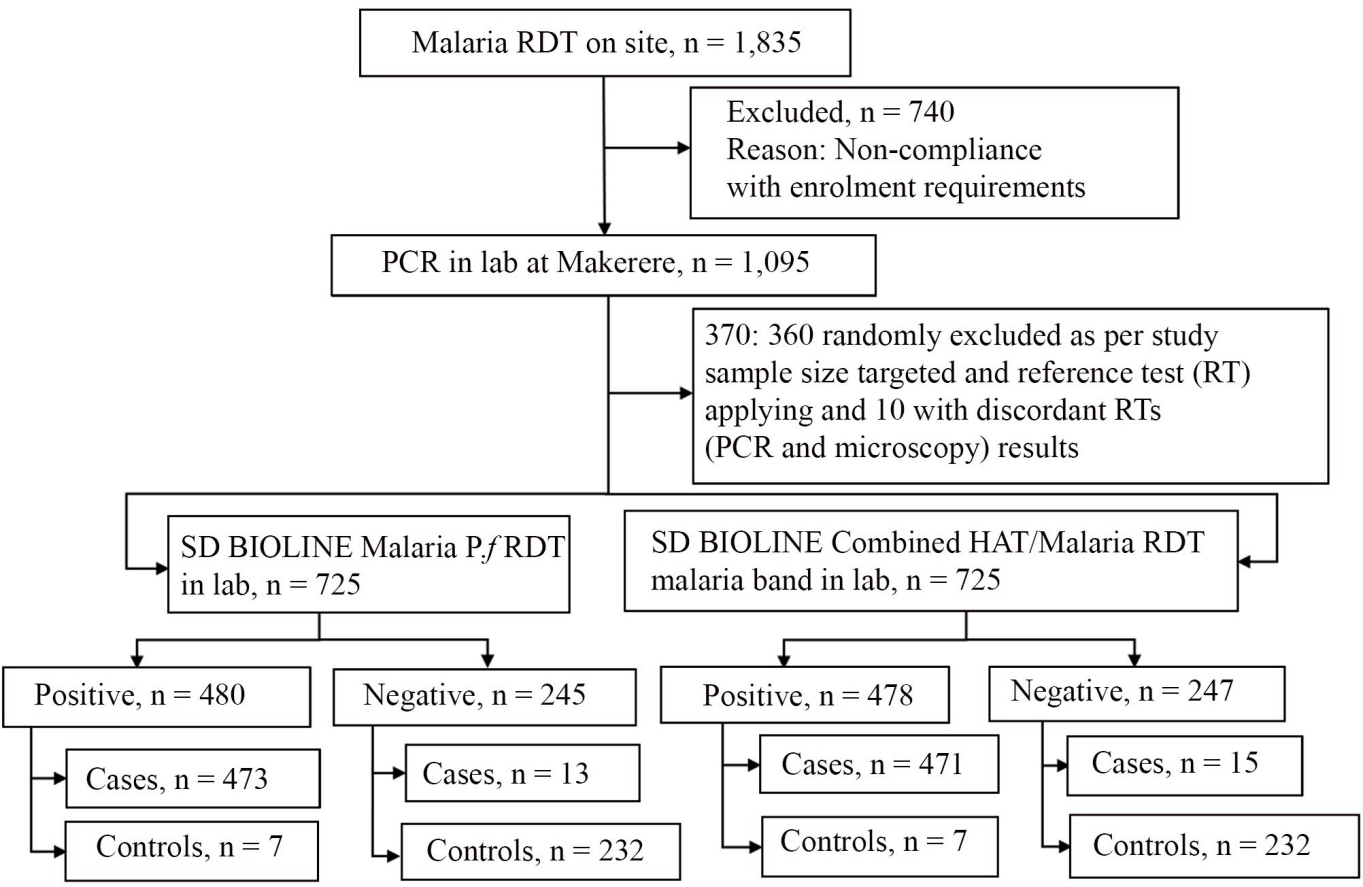
Flow of study participants with regard to malaria assessment.

Among the 725 participants enrolled, the number that tested positive was respectively 480 for individual SD malaria *P*.*f*. RDT, of which 473 were positive with the reference test (RT), 478 positive with the Combo RDT malaria band (471 positive to RT), 245 negative with the individual SD malaria *P*.*f*. RDT (232 negative to RT), and 247 negative with the Combo RDT malaria band (232 negative to RT) as shown in Fig 3.

Among the 480 participants who were positive to the individual malaria *P*.*f*. RDT, 335 were enrolled from HAT endemic regions (333 positive to RT) and 145 (140 positive to RT) from non-HAT regions. Among the 478 participants positive to Combo RDT malaria band, 335 were enrolled from HAT endemic regions (333 positive to RT) and 143 (138 positive to RT) from non-HAT regions.

The number of participants and RDT results related to malaria assessment are detailed in supplemental S1 Table.

#### RDT sensitivity and specificity

The sensitivity and specificity of the RDTs at each site are shown in Table 1. The sensitivity and specificity of the SD Malaria *P*.*f*. RDT were 97.3% (95% CI: 95.5–98.6) and 97.1% (95% CI: 94.1–98.8) respectively. For the malaria band of the Combo RDT, the sensitivity was 96.9% (95% CI: 95.0–98.3) and the specificity was 97.1% (95% CI: 94.1–98.8).

**Table 1:** Malaria sensitivity and specificity of the RDTs by study site.

	SD Malaria *Pf* RDT		Combo RDT malaria band	
Site	Sensitivity (95% CI)	Specificity (95% CI)	Sensitivity (95% CI)	Specificity (95% CI)
**Masamuna**	96.6% (91.4–99.1)	98.7% (92.8–100)	96.6% (91.4–99.1)	98.7% (92.8–100)
**Masimanimba**	97.4% (86.5–99.9)	97.7% (88.0–99.9)	97.4% (86.5–99.9)	97.7% (88.0–99.9)
**Charité Maternelle**	94.4% (81.3–99.3)	93.3% (85.1–97.8)	94.4% (81.3–99.3)	93.3% (85.1–97.8)
**Virunga**	100% (85.8–100)	100% (85.8–100)	95.8% (78.9–99.9)	100% (85.8–100)
**Bethesda**	100% (84.6–100)	100% (83.9–100)	100% (84.6–100)	100% (83.9–100)
**Kasangati**	93.8% (84.8–98.3)	-	92.2% (82.7–97.4)	-
**Omugo**	98.9% (96.1–99.9)	-	98.9% (96.1–99.9)	-

The sensitivity of the SD Malaria *P*.*f*. RDT was of 97.9% (95% CI: 95.8–99.2) in HAT endemic regions, while it was 95.9% (95% CI: 91.3–98.5) in non-HAT endemic regions. The sensitivity of the Combo RDT malaria band was 97.9% (95% CI: 95.8–99.2) in HAT endemic regions and 94.5% (95% CI: 89.5–97.6) in non-HAT endemic regions. The specificity of the SD Malaria *P*.*f*. RDT was 98.3% (95% CI: 94.1–99.8) and 95.8% (95% CI: 90.5–98.6) in HAT endemic and non-HAT endemic regions respectively, while the specificity of the Combo RDT malaria band was 98.3% (95% CI: 94.1–99.8) and 95.8% (95% CI: 90.5–98.6), respectively.

Concordance between the SD malaria *P*.*f*. RDT and the Combo RDT malaria band was 99.4%. Inter-reader agreement for SD Malaria *P*.*f*. RDT and for Combo RDT malaria band was 100.0% and 98.5% respectively.

#### Test accuracy

Youden’s index for accuracy was 0.944 (95% CI: 0.929–0.957) for the individual SD Malaria *P*.*f*. RDT and 0.940 (95% CI: 0.925–0.953) for the Combo RDT malaria band.

#### Predictive values and field application

Considering the prevalence of malaria among febrile patients (prior test probability among febrile outpatients) in DRC (70% in 2016), the RDTs’ positive predictive values (PPV) were 98.7% (95% CI: 97.4–99.4) for both individual SD malaria *P*.*f*. RDT and Combo RDT malaria band. The negative predictive values (NPV) were 94% (95% CI: 90.1–96.4) and 93.1% (95% CI: 89.1–95.7) respectively for the SD individual malaria *P*.*f*. RDT and the Combo RDT malaria band.

### HAT

#### Enrolment

**Fig 4:**
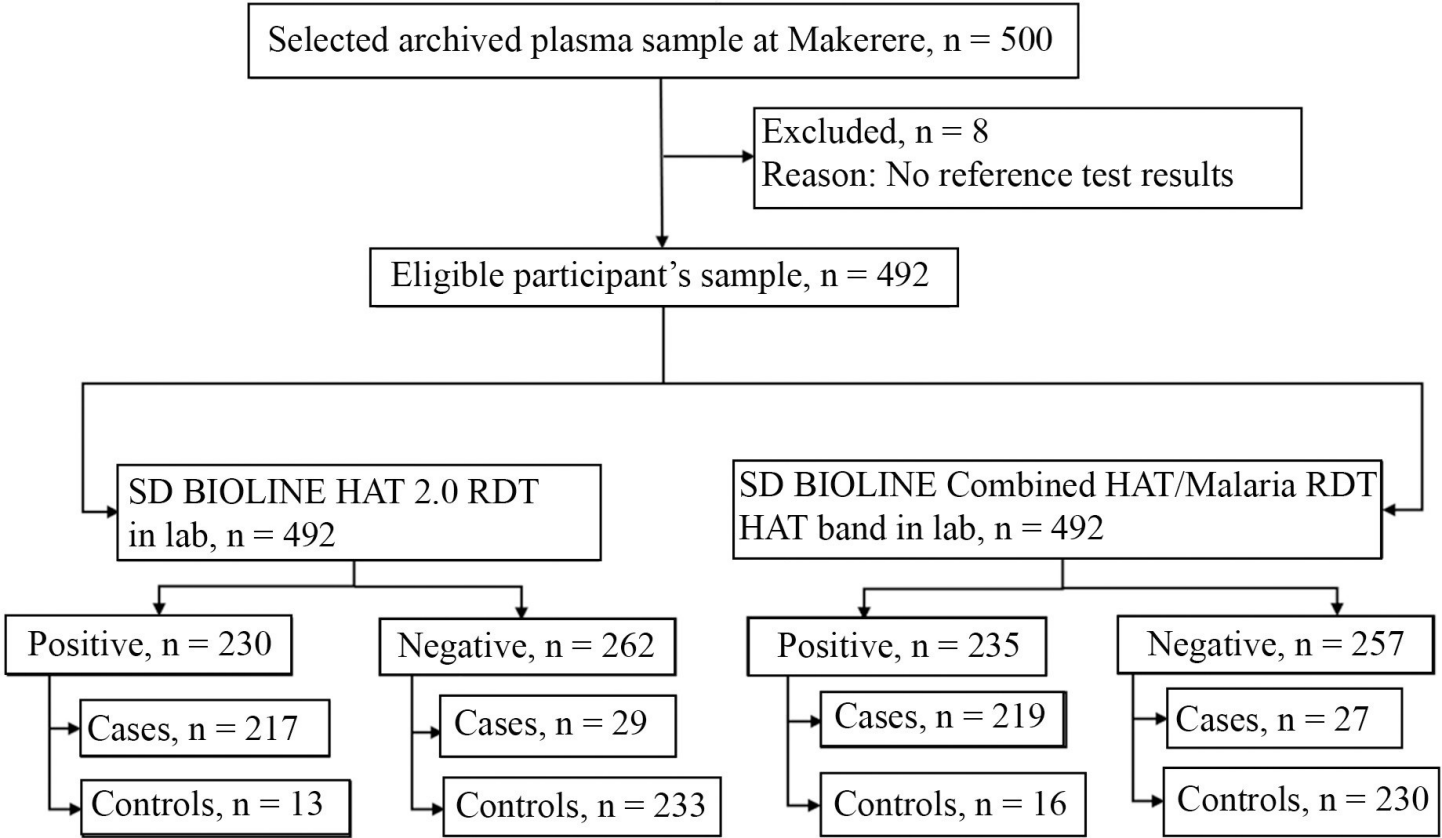
Flow of study participant’s archived plasma samples with regard to HAT assessment.

The number of participants and RDT results per site related to HAT assessment are detailed in S1 Table. A total of 492 archived samples were used, including 246 HAT cases and 246 non-HAT controls. Among the 246 HAT cases, 217 samples were positive with individual HAT 2.0 RDT and 219 with the Combo RDT HAT band. Among 246 non-HAT controls, 233 were negative with individual HAT 2.0 RDT and 230 with the Combo RDT HAT band (Fig 4).

**Fig 5:**
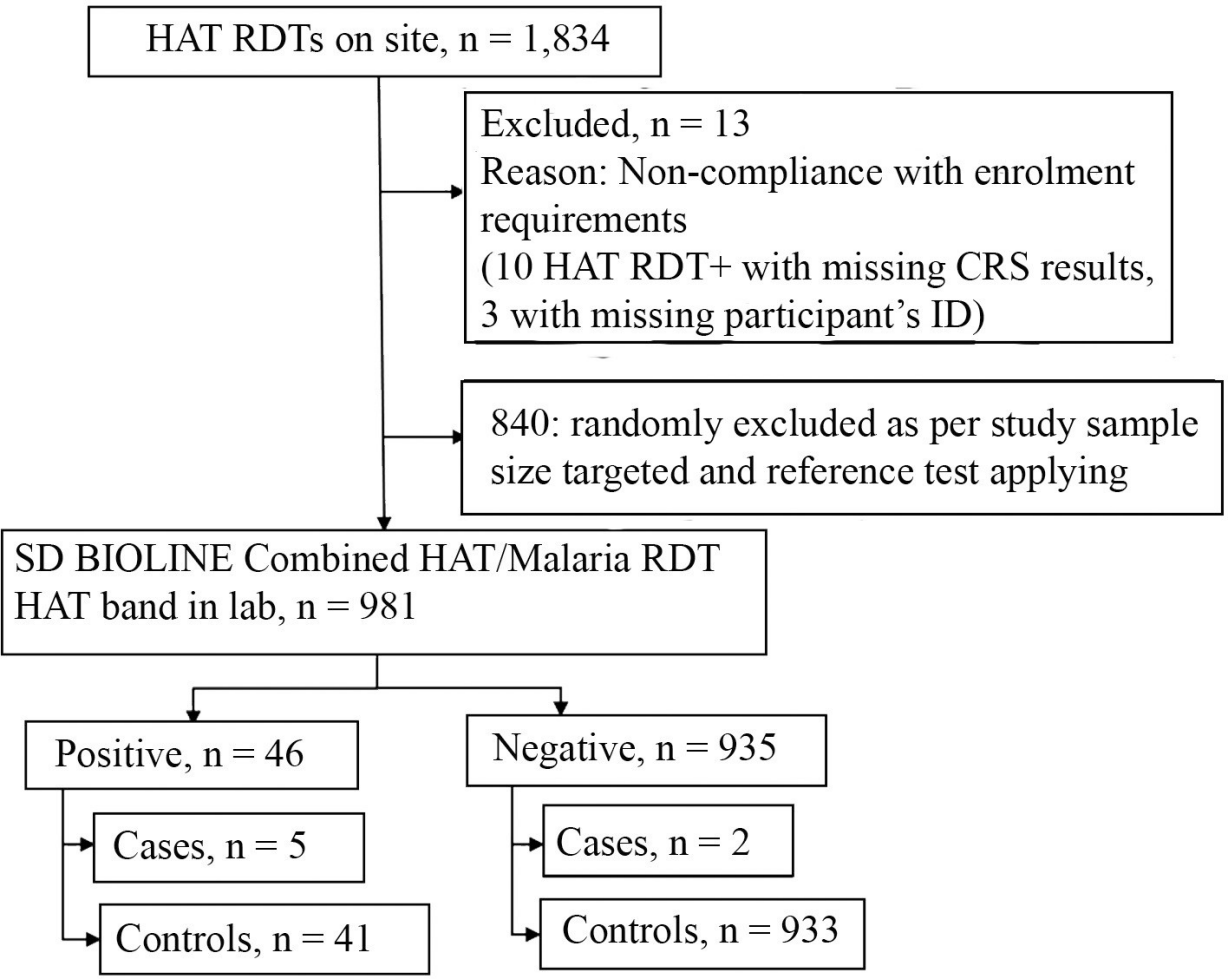
Flow of study participants with regard to HAT assessment using frozen whole blood samples.

The frozen blood samples shipped to Makerere for testing were collected from 981 participants successfully enrolled for HAT assessment. Samples were tested with the Combo RDT (HAT band) and not with the HAT 2.0 RDT. Among the 981 participants, 7 were HAT cases and 974 non-HAT controls, 935 tested negative to the Combo RDT HAT band (933 non-HAT controls) and 46 tested positive (41 non-HAT controls) as shown in Fig 5. In order to assess a potential difference in test accuracy depending on HAT endemicity, 599 participants (including 592 non-HAT controls) were enrolled in HAT endemic regions and 382 (all non-HAT controls) in non-HAT regions. The median age of participants successfully enrolled for HAT assessment was 25 years, with an interquartile range (IQR) of 17 years (19–36 years old). The proportion of females was 63.3% (95% CI: 60.2–66.3).

Among the 935 participants that were negative with the Combo RDT HAT band, 560 were enrolled from HAT endemic regions (558 non-HAT controls) and 375 (375 non-HAT controls) from non-HAT regions. Among the 46 participants that were positive with the Combo RDT HAT band, 39 were enrolled from HAT endemic regions (34 non-HAT controls) and 7 (7 non-HAT controls) from non-HAT regions.

#### RDT sensitivity and specificity

Using archived plasma samples, the sensitivity was 88.2% (95% CI: 83.5–92) and 89.0% (95% CI: 84.4–92.6) for the HAT 2.0 RDT and prototype Combo RDT HAT band respectively. The specificity was 94.7% (95% CI: 91.1–97.2) and 93.5 (95% CI: 89.7–96.2) for the HAT 2.0 RDT and prototype Combo RDT HAT band, respectively.

Concordance between the HAT 2.0 RDT and the prototype Combo RDT HAT band was 91.4%.

The inter-reader agreement was 93.9% and 94.7% for the HAT 2.0 RDT and the prototype Combo RDT HAT band, respectively.

Using frozen blood samples, the overall specificity of the Combo RDT HAT band was 95.8% (95% CI: 94.3–97.0). Its specificity per site is detailed in Table 2 and it was 94.4% (95% CI: 91.9–96.4) in HAT endemic regions and 98.5% (95% CI: 96.3–99.6) in non-HAT endemic regions. The inter-reader agreement was 99.1%.

**Table 2:** Combo RDT HAT band specificity per study site.

	HAT endemic regions			Non-HAT endemic regions			
Site	Masamuna	Masimanimba	Omugo	Charité Maternelle	Virunga	Bethesda	Kasangati
**Specificity (CI 95%)**	96.8% (93.2–98.8)	93.6% (85.7–97.9)	92.3% (87.5–95.8)	97.4% (92.6–99.5)	97.9% (88.9–99.9)	100% (91.8–100)	100% (94.4–100)

#### Test accuracy

Based on archived plasma samples, Youden’s index for accuracy was 0.829 (95% CI: 0.776–0.874) for the HAT 2.0 RDT and 0.825 (95% CI: 0.772–0.871) for the Combo RDT HAT band.

#### Predictive values and field application

The PPV and NPV at 0.01%, 0.1%, 1% and 2% HAT prevalence are detailed in Table 3. Considering the current HAT prevalence of 0.1% in DRC, the RDTs’ PPVs were 1.64% and 1.35%, respectively for HAT 2.0 RDT and the Combo RDT HAT band. The RDTs’ NPV, at the same HAT prevalence rate, were 100% for both HAT 2.0 RDT and the Combo RDT HAT band.

**Table 3:** The Combo RDT and HAT 2.0 RDT predictive values for screening HAT.

HAT Prevalence	0.01%	0.1%	1%	2%
**Combined RDT**				
PPV (%) (95% CI)	0.1 (0.1–0.2)	1.4 (0.8–2.2)	12.1 (7.9–18.2)	21.8 (14.8–31)
NPV (%) (95% CI)	100	100	99.9 (99.8–99.9)	99.8 (99.7–99.8)
**SD HAT 2.0 RDT**				
PPV (%) (95% CI)	0.2 (0.1–0.28)	1.6 (1.0–2.8)	14.4 (9.0–22.3)	25.4 (16.7–36.7)
NPV (%) (95% CI)	100	100	99.9 (99.8–99.9)	99.7 (99.6–99.8)

## Discussion

The present study demonstrated that the HAT/Malaria Combined RDT prototype was as sensitive and specific as the individual SD BIOLINE malaria and HAT RDTs (SD Malaria *P*.*f*. RDT and HAT 2.0 RDT) in the detection of malaria and screening for HAT.

The concordance between the SD BIOLINE HAT/Malaria Combined test and the individual SD Malaria *P*.*f*. RDT and HAT 2.0 RDT was very good.

Malaria RDTs should be selected according to country guidelines, and WHO recommends taking into account the results of the WHO product testing programme for malaria RDTs [6]. The SD Malaria *P*.*f*. RDT that was used as comparator test in the present study has been consistently ranked among the best-performing RDTs in this programme, which is also in line with the WHO/USAID informal consultation that took place in 1999 and stated that malaria RDTs should have a sensitivity above 95% and a specificity of at least 90%. The performance of the SD malaria *P*.*f*. RDT used in the current study was in agreement with WHO recommendations [33].

We obtained a PPV of the evaluated SD malaria *P*.*f*. RDT comparable or higher at similar or lowest malaria prevalence compared to previous studies, while the NPV was higher [26, 34]. The false positive rate found in the current study would result into unwarranted treatment of 1.3 persons out of 100 with either individual SD malaria *P*.*f*. RDT or Combo RDT malaria band positive results, considering a malaria prevalence of 70% (the prior test probability among febrile outpatients in the DRC in 2016). On the other hand, 6 patients suffering from malaria out of one thousand people would be tested negative to the individual SD malaria *P*.*f*. RDT and 7 malaria patients out of one thousand people would be tested negative to the Combo RDT malaria band at the same prevalence.

With regard to HAT screening, Lumbala *et al* (2018) reported an overall HAT 2.0 prototype RDT sensitivity and specificity in both active and passive screening settings of 71.2% (95% CI: 65.7–76.6) and 98.1% (95% CI: 98.0–98.2) respectively. The HAT 2.0 prototype RDT sensitivity and specificity during passive screening, in health facility settings was 90.1% (95% CI: 84.7–95.3) and 93.7% (95% CI: 93.2–94.2) respectively, and 54.8% (95% CI: 46.8–63.5) and 99.1% (95% CI: 99.0–99.2) respectively during active screening by mobile teams [24].

The sensitivity of the HAT 2.0 RDT in the present study was higher than that reported by Lumbala *et al* [24], particularly in active screening (and when combining active and passive screening). This could be explained by the design of the previous study, in which three screening tests were used to identify suspects at the enrolment step (CATT, HAT RDT and HAT 2.0 RDT), while a number of the archived clinical samples used in the current study were collected using one or two screening tests (CATT and/or HAT RDT and/or HAT 2.0 RDT). Using three screening tests in the previous study could have resulted in underestimating the sensitivity of the HAT 2.0 RDT, when cases were only detected by the other screening tests.

The specificity of the HAT 2.0 RDT was significantly lower in the current study than what was reported by Lumbala *et al* (2018), especially in active screening setting (as well as when combining active and passive screening settings). The fact that a number of samples that were used in the current study were collected in CAR without using mAECT, the most sensitive parasitological test for HAT, could have resulted in some HAT cases being missed and wrongly considered as negative in the present study, thus resulting in an underestimate of the RDT specificity.

Due to the relatively low specificity, the false positive rate tends to be high, especially now that HAT prevalence has decreased significantly, especially in active screening settings. With 0.1% HAT prevalence, at least 98 people out of 100 screened positive to HAT 2.0 RDT or Combo RDT HAT band would be false positive. At 1% HAT prevalence, only 14 persons out of 100 screened positive to HAT 2.0 RDT and 12 persons out of 100 screened positive to Combo RDT HAT band would be confirmed as HAT cases. Currently, a HAT prevalence of 1% can only be achieved in passive screening, and rarely in active screening. On the other hand, for every 1,000 negative screening results, 1 and 2 would be false negatives (missed cases) with Combo RDT HAT band, compared to 1 and 3 false negatives with HAT 2.0 RDT respectively at 1% and 2% HAT prevalence. As the PPV is declining rapidly with current prevalence and the progress towards HAT elimination, this will result in an increase in workload to confirm suspects, with a negative impact on health workers’ and patients’ motivation and confidence in positive test results, which would end up in missing true HAT cases. The combo RDT is intended to be used primarily for malaria testing and particularly by malaria programs in HAT endemic settings. But based on the PPV at the current HAT prevalence, it seems that while integration of HAT screening in the malaria control program is theoretically meant to increase the number of HAT tests done and therefore increase the proportion of HAT cases detected, the low PPV might quickly have a negative impact on use of the combo RDT. Indeed, introduction of the combo RDT may lead to HAT testing in sites where the HAT prevalence is even lower than 0.1% (0.01% being the actual cut-off for elimination of HAT as a public health problem). The steady decline in HAT prevalence is leading to a decrease in the positive predictive value, while all persons identified as HAT serological suspects need parasitological confirmation by dedicated personnel. In the context of being confronted with a vast majority of false positives, logistic and financial limitations to perform parasitological HAT confirmation, there is a risk that malaria program health personnel will quickly reject the Combo RDT and return to a single malaria test. This underlines the potential shortcoming of the Combo RDT. To prevent this, it will be necessary to improve the PPV of HAT screening RDTs by for instance increasing the pre-test probability by restraining the use of the Combo RDTs to patients with clinical signs that are more specific of HAT, or using an algorithm that includes more specific molecular or serological tests. As test line positivity for malaria (antigen detection) and HAT (antibody detection) may have a different meaning, in particular for treatment decision, training, follow-up and supervision will be very important prior to introduction of the combo RDT in health facilities and / or its integration in malaria programs.

### Limitations of this study

The current study had two main limitations that prevent further analysis and interpretation of results. First, information related to possible malaria treatment up to 42 days prior to blood collection was not available. False positivity following a successful malaria treatment would result in an underestimated RDT specificity. The fact that this information was not collected limits comparison to other study findings, especially in regard of malaria RDT specificity.

Second, due to the continued decline in HAT prevalence, it was not possible to evaluate RDT sensitivity using freshly collected blood, but only using archived plasma. The archived plasma samples had been pre-selected using various screening tests, and some of the samples were rather old (2008) albeit well stored in -80°C freezers or in liquid nitrogen. This could have had an impact on our results and would therefore make comparisons with other studies difficult. However, the fact that the current study aimed to compare the performance of RDTs using the same samples and in the same testing conditions still makes our findings relevant.

Finally, the fresh blood samples that were collected and shipped frozen to Makerere University were not tested with the HAT 2.0 RDT. It was therefore not possible to compare the HAT specificity of RDTs.

### Conclusion

This study has demonstrated equivalence in sensitivity and specificity of a prototype HAT/Malaria Combined RDT for both HAT and malaria, in comparison to the individual HAT and malaria RDTs.

The HAT/Malaria Combined RDT could be used to enable an integrated approach combining malaria diagnosis and HAT screening in *gambiense* HAT endemic areas. A similar strategy would be a good solution to the increasing problem of under-detection of *rhodesiense* HAT (rHAT), but a rapid diagnostic test for rHAT has so far not been developed. However, this evaluation was performed in an academic laboratory setting which differs from field conditions. Also, testing was done on frozen and/or archived samples, which may have slightly different results to those that would be obtained using fresh blood samples. Thus, there is need to conduct prospective clinical trials to confirm the performance of the HAT/Malaria Combined test under real field conditions and assess the accuracy in the local laboratory at the level of health facilities.

## Supporting information

S1 Checklist STARD checklist. (PDF)

S1 Table Number of samples and results of malaria assessment per site and per test. (DOCX)

S2 Table Number of samples and results of HAT assessment per site and per test. (DOCX)

S1 Data Combo RDT HAT and malaria assessment using frozen whole blood sample data base. (CSV)

S2 Data Combo RDT HAT assessment using archived plasma sample data base. (CSV)
