# Supplementary material for: Performance evaluation of a prototype rapid diagnostic test for combined detection of gambiense human African trypanosomiasis and malaria
Source: PLoS Negl Trop Dis. 2020 Apr 6;14(4):e0008168. doi: 10.1371/journal.pntd.0008168 (PMC7162526; doi:10.1371/journal.pntd.0008168)
Supplement: S1 Checklist — (PDF) [file pntd.0008168.s001.pdf]

| Section & Topic | No | Item | Reported on page # |
|---|---|---|---|
| **TITLE OR ABSTRACT** | | | |
| | **1** | Identification as a study of diagnostic accuracy using at least one measure of accuracy<br>(such as sensitivity, specificity, predictive values, or AUC) | Title: “Performance evaluation of a prototype combined Rapid Diagnostic Test for Human African Trypanosomiasis and Malaria” |
| **ABSTRACT** | | | |
| | **2** | Structured summary of study design, methods, results, and conclusions<br>(for specific guidance, see STARD for Abstracts) | Abstract: Background, methods, results and conclusion |
| **INTRODUCTION** | | | |
| | **3** | Scientific and clinical background, including the intended use and clinical role of the index test | Introduction (Page 5 – 7) |
| | **4** | Study objectives and hypotheses | Introduction, the 2 last paragraphs (line 116 to 137) |
| **METHODS** | | | |
| *Study design* | **5** | Whether data collection was planned before the index test and reference standard were performed (prospective study) or after (retrospective study) | Retrospective Study |
| *Participants* | **6** | Eligibility criteria | Methods, “Enrolment of participants for sample collection” section |
| | **7** | On what basis potentially eligible participants were identified<br>(such as symptoms, results from previous tests, inclusion in registry) | Methods, “Enrolment of participants for sample collection” section<br>- Clinical suspicion (Fever, headache, flue-like syndrome…) |
| | **8** | Where and when potentially eligible participants were identified (setting, location and dates) | Methods, “study design” section:<br>- For malaria: Masamuna, Masimanimba Bethesda, Virunga and Charité Maternelle Hospitals in DRC, from 21 November 2016 to 11 May 2017, and Kasangati and Omugo IV Health Centres in Uganda<br>- For HAT: Angola, CAR and DRC, archived storage sample (2008, 2015, 2016) |
| | **9** | Whether participants formed a consecutive, random or convenience series | Consecutive |
| *Test methods* | **10a** | Index test, in sufficient detail to allow replication | Methods, “HAT and malaria RDTs” section:<br>The SD BIOLINE HAT/Malaria Combined prototype RDT includes one control band (C band) two test bands: one to detect the *P. falciparum* HRP-II antigen (“P.f or malaria” band) and the other to detect antibodies against two trypanosome antigens (recombinant ISG65 and VSG LiTat 1.5) (“HAT” band). |
| | **10b** | Reference standard, in sufficient detail to allow replication | Methods, “Enrolment of participants for sample collection” section:<br>- For malaria: microscopy (performed in the field on fresh blood samples) and Polymerase Chain Reaction (performed at Makerere University on stored samples). The sample was excluded if microscopy and PCR results were discordant.<br>- For HAT: composite reference test made of microscopy in either blood, lymph node aspirate or cerebrospinal fluid |
| | **11** | Rationale for choosing the reference standard (if alternatives exist) | Methods, “Enrolment of participants for sample collection” section<br>- For malaria: microscopy combined with PCR is more accurate to diagnose malaria. Many researchers used this combination as reference diagnosing test, some used microscopy or PCR solely as reference test. On our side, we preferred the combination<br>- For HAT: we use the composite reference test that include the most sensitive test that could be performed in the field. |
| | **12a** | Definition of and rationale for test positivity cut-offs or result categories | Methods, “HAT and malaria RDTs” section: Index test is positive when the malaria or HAT test band turned positive (coloured) |

cc BY SA

| | | | |
|---|---|---|---|
| | | of the index test, distinguishing pre-specified from exploratory | |
| | **12b** | Definition of and rationale for test positivity cut-offs or result categories of the reference standard, distinguishing pre-specified from exploratory | Methods, "Enrolment of participants for sample collection" section:<br>- for malaria: reference standard is positive when both microscopy and PCR are positive<br>- for HAT: composite reference test is positive when trypanosome is found in body fluid |
| | **13a** | Whether clinical information and reference standard results were available to the performers/readers of the index test | Methods, "Collection and use of blood samples "and "Evaluation of RDTs using stored clinical samples" section, 1st paragraph (Clinical information and reference standard results were not available to Index tests performers/readers) |
| | **13b** | Whether clinical information and index test results were available to the assessors of the reference standard | Methods, "Enrolment of participants for sample collection" section, 3rd paragraph, "Collection and use of blood samples" section, and "Microscopy procedures and PCR for malaria" paragraph (Clinical information and reference standard results were not available to assessors of the reference standard) |
| *Analysis* | **14** | Methods for estimating or comparing measures of diagnostic accuracy | Methods, "Data management and statistical analysis": Sensitivity (positive index tests among cases), specificity (negative index tests among controls), Youden's index |
| | **15** | How indeterminate index test or reference standard results were handled | Methods, "Enrolment of participants for sample collection" section (They were excluded) |
| | **16** | How missing data on the index test and reference standard were handled | Participants with missing data on the index test or reference standard were excluded from the analysis. |
| | **17** | Any analyses of variability in diagnostic accuracy, distinguishing pre-specified from exploratory | Not applicable |
| | **18** | Intended sample size and how it was determined | Methods, "Enrolment of participants for sample collection" section:, last paragraph: no formal sample size calculations were performed, but the number of samples targeted was expected to provide information that will be strong enough to evaluate prototype and make decisions to start prospective performance evaluation studies in the field. |
| **RESULTS** | | | |
| *Participants* | **19** | Flow of participants, using a diagram | Supplementary Information File 2 |
| | **20** | Baseline demographic and clinical characteristics of participants | Results, malaria section, enrolment sub-section and HAT section, enrolment subsection. |
| | **21a** | Distribution of severity of disease in those with the target condition | Methods, "Enrolment of participants for sample collection" section:<br>- paragraph 1: Patients with severe conditions like anaemia preventing blood collection or in conditions preventing administration of the informed consent were excluded from the study. Just clinical signs and symptoms with suspect to HAT or malaria was considered.<br>- Paragraph 3: Patients with known history of previous HAT infection were excluded from HAT controls. |
| | **21b** | Distribution of alternative diagnoses in those without the target condition | Not applicable |
| | **22** | Time interval and any clinical interventions between index test and reference standard | Methods,<br>"study design" section: To evaluate the accuracy of HAT screening, plasma samples obtained from HAT endemic countries (Angola, CAR and DRC) as part of earlier research projects (2008, 2015, 2016) and archived at Makerere University (Kampala, Uganda) were used.<br>"Enrolment of participants for sample collection" section: Different tests were performed blindingly. Microscopy was performed in the field, PCR and the index test (RDT) at the same time in lab at Makerere University. The interval between microscopy and PCR/index test was between 3 and 9 months depending on the date of sample collection and transfer to Makerere University laboratory. |
| *Test results* | **23** | Cross tabulation of the index test results (or their distribution) by the results of the reference standard | This information is included in the text (Results, malaria section enrolment subsection and HAT section and enrolment subsection) and in Fig 2. |

CC BY SA

| | | | |
|---|---|---|---|
| | **24** | Estimates of diagnostic accuracy and their precision (such as 95% confidence intervals) | Results, malaria section and “RDT sensitivity and specificity” and “test accuracy” subsections; HAT section and “RDT sensitivity and specificity” and “test accuracy” subsections. |
| | **25** | Any adverse events from performing the index test or the reference standard | No adverse events were reported |
| **DISCUSSION** | | | |
| | **26** | Study limitations, including sources of potential bias, statistical uncertainty, and generalisability | Discussion, paragraph 3 (If persistence of HRP-2 antigen in blood in some study participant that experienced prior infection and are effectively treated could explain this low specificity, this has not been checked in our study.)<br>And the paragraph 6: the design of the previous study where three screening tests were used to identify suspects at the enrolment step, while the clinical samples used in the current study came from various projects following procedures that were quite different; samples collected in Angola and CAR were pre-selected by CATT, and the ones collected in DRC were pre-selected by CATT and 2 different HAT RDTs, while some samples from Angola had been in storage for a long time (collected in 2008). |
| | **27** | Implications for practice, including the intended use and clinical role of the index test | Conclusion, 2nd paragraph: To facilitate integration between HAT screening and malaria diagnosis and therefore help to find the last HAT cases while testing for malaria more prevalent |
| **OTHER INFORMATION** | | | |
| | **28** | Registration number and name of registry | |
| | **29** | Where the full study protocol can be accessed | From FIND, Geneva |
| | **30** | Sources of funding and other support; role of funders | BMGF<br>The funders had no role in study design, data collection and analysis, decision to<br>publish, or preparation of the manuscript. |

# STARD 2015

## AIM

STARD stands for “Standards for Reporting Diagnostic accuracy studies”. This list of items was developed to contribute to the completeness and transparency of reporting of diagnostic accuracy studies. Authors can use the list to write informative study reports. Editors and peer-reviewers can use it to evaluate whether the information has been included in manuscripts submitted for publication.

## EXPLANATION

A **diagnostic accuracy study** evaluates the ability of one or more medical tests to correctly classify study participants as having a **target condition.** This can be a disease, a disease stage, response or benefit from therapy, or an event or condition in the future. A medical test can be an imaging procedure, a laboratory test, elements from history and physical examination, a combination of these, or any other method for collecting information about the current health status of a patient.

The test whose accuracy is evaluated is called **index test.** A study can evaluate the accuracy of one or more index tests. Evaluating the ability of a medical test to correctly classify patients is typically done by comparing the distribution of the index test results with those of the **reference standard**. The reference standard is the best available method for establishing the presence or absence of the target condition. An accuracy study can rely on one or more reference standards.

If test results are categorized as either positive or negative, the cross tabulation of the index test results against those of the reference standard can be used to estimate the **sensitivity** of the index test (the proportion of participants *with* the target condition who have a positive index test), and its **specificity** (the proportion *without* the target condition who have a negative index test). From this cross tabulation (sometimes referred to as the contingency or “2x2” table), several other accuracy statistics can be estimated, such as the positive and negative **predictive values** of the test. Confidence intervals around estimates of accuracy can then be calculated to quantify the statistical **precision** of the measurements.

If the index test results can take more than two values, categorization of test results as positive or negative requires a **test positivity cut-off**. When multiple such cut-offs can be defined, authors can report a receiver operating characteristic (ROC) curve which graphically represents the combination of sensitivity and specificity for each possible test positivity cut-off. The **area under the ROC curve** informs in a single numerical value about the overall diagnostic accuracy of the index test.

The **intended use** of a medical test can be diagnosis, screening, staging, monitoring, surveillance, prediction or prognosis. The **clinical role** of a test explains its position relative to existing tests in the clinical pathway. A replacement test, for example, replaces an existing test. A triage test is used before an existing test; an add-on test is used after an existing test.

Besides diagnostic accuracy, several other outcomes and statistics may be relevant in the evaluation of medical tests. Medical tests can also be used to classify patients for purposes other than diagnosis, such as staging or prognosis. The STARD list was not explicitly developed for these other outcomes, statistics, and study types, although most STARD items would still apply.

## DEVELOPMENT

This STARD list was released in 2015. The 30 items were identified by an international expert group of methodologists, researchers, and editors. The guiding principle in the development of STARD was to select items that, when reported, would help readers to judge the potential for bias in the study, to appraise the applicability of the study findings and the validity of conclusions and recommendations. The list represents an update of the first version, which was published in 2003.

More information can be found on http://www.equator-network.org/reporting-guidelines/stard.

CC BY SA
