# Supplementary material for: Performance evaluation of a prototype rapid diagnostic test for combined detection of gambiense human African trypanosomiasis and malaria
Source: PLoS Negl Trop Dis. 2020 Apr 6;14(4):e0008168. doi: 10.1371/journal.pntd.0008168 (PMC7162526; doi:10.1371/journal.pntd.0008168)
Supplement: S1 Table — (DOCX) [file pntd.0008168.s002.docx]

Supplemental Table S1: Number of samples and results of malaria assessment per site and per test

| **Study site** | **Negative SD Malaria *P.f.* RDT** | **Positive SD Malaria *P.f.* RDT** | **Total** | **Negative Combo RDT malaria band** | **Positive Combo RDT malaria band** | **Total** |
| --- | --- | --- | --- | --- | --- | --- |
| **Masamuna, HAT endemic region, DRC** | | | | | | |
| - Control | 74 | 1 | 75 | 74 | 1 | 75 |
| - Case | 4 | 112 | 116 | 4 | 112 | 116 |
| - Total | 78 | 113 | 191 | 78 | 113 | 191 |
| **Masimanimba, HAT endemic region, DRC** | | | | | | |
| - Control | 43 | 1 | 44 | 43 | 1 | 44 |
| - Case | 1 | 38 | 39 | 1 | 38 | 39 |
| - Total | 44 | 39 | 83 | 44 | 39 | 83 |
| **Omugo, HAT endemic region, Uganda** | | | | | | |
| - Control | 0 | 0 | 0 | 0 | 0 | 0 |
| - Case | 2 | 183 | 185 | 2 | 183 | 185 |
| - Total | 2 | 183 | 185 | 2 | 183 | 185 |
| **HAT endemic regions** |  |  |  |  |  |  |
| - Control | 117 | 2 | 119 | 117 | 2 | 119 |
| - Case | 7 | 333 | 340 | 7 | 333 | 340 |
| - Total | 124 | 335 | 459 | 124 | 335 | 459 |
| **Charité Maternelle, non- HAT endemic region, DRC** | | | | | | |
| - Control | 70 | 5 | 75 | 70 | 5 | 75 |
| - Case | 2 | 34 | 36 | 2 | 34 | 36 |
| - Total | 72 | 39 | 111 | 72 | 39 | 111 |
| **Virunga, non- HAT endemic region, DRC** | | | | | | |
| - Control | 24 | 0 | 24 | 24 | 0 | 24 |
| - Case | 0 | 24 | 24 | 1 | 23 | 24 |
| - Total | 24 | 24 | 48 | 25 | 23 | 48 |
| **Bethesda, non- HAT endemic region, DRC** | | | | | | |
| - Control | 21 | 0 | 21 | 21 | 0 | 21 |
| - Case | 0 | 22 | 22 | 0 | 22 | 22 |
| - Total | 21 | 22 | 43 | 21 | 22 | 43 |
| **Kasangati, non- HAT endemic region, Uganda** | | | | | | |
| - Control | 0 | 0 | 0 | 0 | 0 | 0 |
| - Case | 4 | 60 | 64 | 5 | 59 | 64 |
| - Total | 4 | 60 | 64 | 5 | 59 | 64 |
| **Non-HAT endemic regions** | | | | | | |
| - Control | 115 | 5 | 120 | 115 | 5 | 120 |
| - Case | 6 | 140 | 146 | 8 | 138 | 146 |
| - Total | 121 | 145 | 266 | 123 | 143 | 266 |
