# Supplementary material for: Performance evaluation of a prototype rapid diagnostic test for combined detection of gambiense human African trypanosomiasis and malaria
Source: PLoS Negl Trop Dis. 2020 Apr 6;14(4):e0008168. doi: 10.1371/journal.pntd.0008168 (PMC7162526; doi:10.1371/journal.pntd.0008168)
Supplement: S2 Table — (DOCX) [file pntd.0008168.s003.docx]

S2 Table: Number of samples and results of HAT assessment per site and per test

| **Study site** | **Negative Combo RDT HAT band** | **Positive Combo RDT HAT band** | **Total** |
| --- | --- | --- | --- |
| **Masamuna** |  |  |  |
| - Control | 185 | 6 | 191 |
| - Case | 0 | 2 | 2 |
| - Total | 185 | 8 | 193 |
| **Masimanimba** |  |  |  |
| - Control | 73 | 5 | 78 |
| - Case | 2 | 3 | 5 |
| - Total | 75 | 8 | 83 |
| **Charité Maternelle** |  |  |  |
| - Control | 113 | 3 | 116 |
| - Case | 0 | 0 | 0 |
| - Total | 113 | 3 | 116 |
| **Virunga** |  |  |  |
| - Control | 47 | 1 | 48 |
| - Case | 0 | 0 | 0 |
| - Total | 47 | 1 | 48 |
| **Bethesda** |  |  |  |
| - Control | 43 | 0 | 43 |
| - Case | 0 | 0 | 0 |
| - Total | 43 | 0 | 43 |
| **Kasangati** |  |  |  |
| - Control | 172 | 3 | 175 |
| - Case | 0 | 0 | 0 |
| - Total | 172 | 3 | 175 |
| **Omugo** |  |  |  |
| - Control | 300 | 23 | 323 |
| - Case | 0 | 0 | 0 |
| - Total | 300 | 23 | 323 |
